# A New Demodecidae Mite (Acariformes: Prostigmata) Parasitizing the Raccoon *Procyon lotor* (Carnivora: Procyonidae), an Invasive Species in Europe †

**DOI:** 10.3390/insects16121218

**Published:** 2025-11-28

**Authors:** Joanna N. Izdebska, Leszek Rolbiecki, Katarzyna Faleńczyk-Koziróg, Dariusz J. Gwiazdowicz

**Affiliations:** 1 Department of Invertebrate Zoology and Parasitology, Faculty of Biology, University of Gdańsk, Wita Stwosza 59, 80-308 Gdańsk, Poland; leszek.rolbiecki@ug.edu.pl (L.R.); katarzyna.falenczyk-kozirog@ug.edu.pl (K.F.-K.); 2 Department of Forest Entomology and Pathology, University of Life Sciences, Wojska Polskiego 71c, 60-625 Poznań, Poland

**Keywords:** biological invasions, *Demodex procyonis*, demodecid mite, infestation, mammals, parasites, procyonids

## Abstract

The raccoon, *Procyon lotor*, is an alien species in Europe, where it is considered invasive and poses significant environmental and sanitary risks: it can increase the host reservoir by both introducing new parasites to the region and acquiring native ones. One permanent component of the mammalian parasitofauna is the Demodecidae, highly specific skin mites that often accompany their hosts in various regions of their range. To date, unidentified *Demodex* species have been found in raccoons within their native range. It has now become important to determine whether raccoons from invasive populations retain their specific parasites during range expansion and potentially spread them or whether they become colonized by other demodecid mites acquired from European mammals. The present analyses of three raccoons identified *Demodex procyonis* sp. nov. in the skin. This species is distinct from demodecid mites known from other mammals (including European species) but shows similarities in structure and location to mites found on raccoons in the United States. It is therefore likely to be a raccoon-specific species of Demodecidae and the first representative of this mite family described from the family Procyonidae.

## Simple Summary

Mites from the Demodecidae family are specific skin parasites of mammals and typically accompany their hosts within their natural geographic range. Monitoring the occurrence of these parasites in mammals expanding into new areas is of particular interest. Currently, the raccoon, an American carnivoran considered an alien and invasive species in Europe, is under study. *Demodex procyonis* sp. nov. has been described in individuals from Poland and appears to be identical to an unknown species of demodecid mite found in this host in its native range in the USA. This confirms the possibility of maintaining the natural parasitofauna typical of the host, as well as the introduction and possible spread of parasites to new regions.

## 1. Introduction

The raccoon, *Procyon lotor* (Linnaeus, 1758) (Carnivora, Procyonidae), is an alien and invasive species in Europe and poses significant environmental and sanitary threats [1,2,3]. Following its introduction to Europe in the first half of the 20th century, when it was first released into the wild in Germany in the 1930s and 1940s, the raccoon has gradually spread across the continent, with a rapid expansion noted since the 1980s. While studies of population genetics confirm that the Central European raccoon population originated from the initial German release sites, further individual releases have also taken place in other European countries. From their Central European origin, the original populations spread widely, mainly to Germany, Denmark, the Netherlands, Belgium, Luxembourg, and France. In Poland, raccoons began to appear sporadically after 1945 following escapes from breeding farms, and wild populations have been recorded since around 1990. The populations in the western part of the country tend to be more stable, and while they have likely already colonized almost the entire country, their expansion into the rest of Poland has been slower. Nevertheless, due to their high ecological plasticity, raccoons are able to both colonize forest areas and expand into urbanized environments [1,2,4]. Like other alien species, they can serve both as sources of new parasites introduced into a region and as additional reservoirs for native parasites and pathogens. The parasitofauna of alien species is a dynamic system. This is because such communities may include parasite species that form part of the natural parasitofauna in their native range and have been secondarily introduced into new regions together with their hosts. There, they may persist or undergo a gradual loss [5,6]. Hosts may also gradually acquire new, local parasites, increasing the reservoir of their occurrence [7,8,9]. Therefore, parasites transmitted by alien species may change or disrupt interactions between species; these interactions may be shaped in long-term evolutionary processes by natural selection, including existing host–parasite relationships [10]. The presence of specific parasites is of great importance. These are usually well-tolerated, often asymptomatic forms and can be transmitted together with their hosts to new regions [11,12]. They may pose a potential threat to local fauna if they find alternative hosts with very similar physiological or ecological traits. In such cases, they may become considerably more pathogenic to the new host [13]. Conversely, their absence may increase the reservoir for other species with similar niches by creating vacant microhabitats [9].

The Demodecidae are highly host-specific skin mites that form a permanent component of the mammalian parasitofauna and often accompany their hosts throughout their distribution range [9,14]. To date, there are no available data regarding the potential transfer of these typically monoxenic parasites to other host species, although this cannot be completely ruled out. They are difficult to detect and identify [14]. However, their absence in the skin of a typical host may create opportunities for other skin parasites to colonize vacant microhabitats, potentially leading to an expansion of host range and, consequently, of the reservoir species. To date, an unidentified mite, *Demodex* sp., has been found in raccoons, causing disease symptoms within the natural range of the host [15]. It is therefore important to determine whether these host-specific parasites are retained, and thus spread, by raccoons while expanding their range, or whether they are replaced by other demodicid mites acquired from European mammals.

## 2. Materials and Methods

Three raccoons (♀/9.8 kg, ♀/9.9 kg, ♂/6.8 kg) from Poland (Przytok Forest District, 52°01′49.1′′, 15°33′40.8′′ E), collected in November 2024, were examined for demodecid mites.

The host skin was subjected to fragment digestion to recover skin mites [16]. Skin fragments of 1 cm^2^ were collected from several body regions, including the head (around eyes, nose, area of vibrissae, lips, chin, cheeks, and vertex), neck, abdomen, back, limbs, and genital–anal area. Samples were preserved in 70% ethanol and subjected to digestion in 10% KOH solution; samples obtained were decanted (examination of 1 cm^2^ of the skin is equal to the analysis of approximately 100 wet preparations, i.e., in the liquid state) and analyzed using phase-contrast microscopy (Nikon Eclipse 50i, Tokyo, Japan). The mites were mounted in polyvinyl-lactophenol solution. All measurements are in micrometers and were taken as follows: total body length = length of gnathosoma, podosoma, and opisthosoma; gnathosomal width = width at base; podosomal and opisthosomal widths = maximum width.

The specimen depositories are cited using the following abbreviation: UGDIZP, University of Gdańsk, Department of Invertebrate Zoology and Parasitology, Gdańsk, Poland [17].

The species was morphologically described based on the nomenclature commonly used for the family Demodecidae [18]; this was completed with the nomenclature proposed by Bochkov [19] for the superfamily Cheyletoidea (Acariformes: Prostigmata) and by Izdebska and Rolbiecki [20]. Unfortunately, no molecular analyses have been possible, as the KOH used to digest mites from skin tissues for analysis degrades the genetic material.

The scientific and common names of the hosts follow Wilson and Reeder [21] and the Integrated Taxonomic Information System [22].

## 3. Results

The research identified a new species in the raccoon, *Demodex procyonis* sp. nov., described based on specimens of both sexes.

### 3.1. Descriptions of New Species (Table 1, Figure 1 and Figure 2)

*Demodex* Owen, 1843

*Demodex procyonis* sp. nov. Izdebska, Rolbiecki, Faleńczyk-Koziróg et Gwiazdowicz

Female (n = 17 paratypes and 1 holotype). Body elongated, cylindrical, slender; 193 (178–207) long and 32 (28–36) wide (holotype, 190 × 31). Gnathosoma clearly separated from podosoma; oval, barrel-shaped, with length similar to width at base; on the dorsal side in the central part of the basal segment, a pair of small, pointed (spike) supracoxal spines (setae *elc.p*) present, ca. 2.5–3 long (holotype, 3), directed medially. Palps 3-segmented, terminating in three spines (one small, pointed, unbifurcated, curved, and two larger, including one bifurcated) on the tibiotarsus. On the ventral surface of the gnathosoma, a horseshoe-shaped pharyngeal bulb with a pair of small subgnathosomal setae (setae *n*) is situated clearly above the anterior on both sides. Podosoma cylindrical; four pairs of short legs, with coxa integrated into ventral idiosomal wall and five free, overlapping segments (trochanter–tarsus); two bifurcated claws, ca. 5–6 long (holotype, 5), with sharp spur and large bulge on each tarsus; additionally, two tubercles present at base of each claw. Epimeral plates (coxal fields) I–III are rectangular, distinctly sclerotized, and connected medially; plate IV is weakly sclerotized. On the dorsal side of the podosoma, a podosomal shield is present, reaching the level of legs III. Opisthosoma elongated, cylindrical, gradually tapered towards the end, rounded at the end; constitutes 59% (56–62%) of body length (holotype, 57%). Whole opisthosoma delicately annulated; annuli relatively wide at ca. 3–4. Opisthosomal organ not visible. Vulva 8 (6–9) long (holotype, 7), located between incisions of IV epimeral plates.

**Table 1:** Body size (micrometers) of adults and deutonymphs of *Demodex procyonis* sp. nov.

Morphologic Features	Males (n = 6) Mean (Range) ± SD	Females (n = 18) Mean (Range) ± SD	Deutonymphs (n = 2) Mean (Range) ± SD
Length of gnathosoma	20 (19–21) ± 1	20 (18–21) ± 1	17 (17) ± 0
Width of gnathosoma (at base)	20 (16–22) ± 2	20 (18–21) ± 1	18 (15–21) ± 4
Length of podosoma	57 (49–66) ± 7	59 (55–62) ± 1	44 (43–44) ± 1
Width of podosoma	32 (29–36) ± 3	32 (28–36) ± 2	24 (21–27) ± 4
Length of opisthosoma	89 (78–99) ± 10	114 (100–129) ± 8	105 (102–107) ± 4
Width of opisthosoma	31 (27–35) ± 3	31 (27–36) ± 2	21 (19–23) ± 3
Aedeagus	21 (19–24) ± 2	–	–
Vulva	–	8 (6–9) ± 1	–
Total length of body	166 (147–185) ± 18	193 (178–207) ± 9	165 (162–168) ± 4

**Figure 1:**
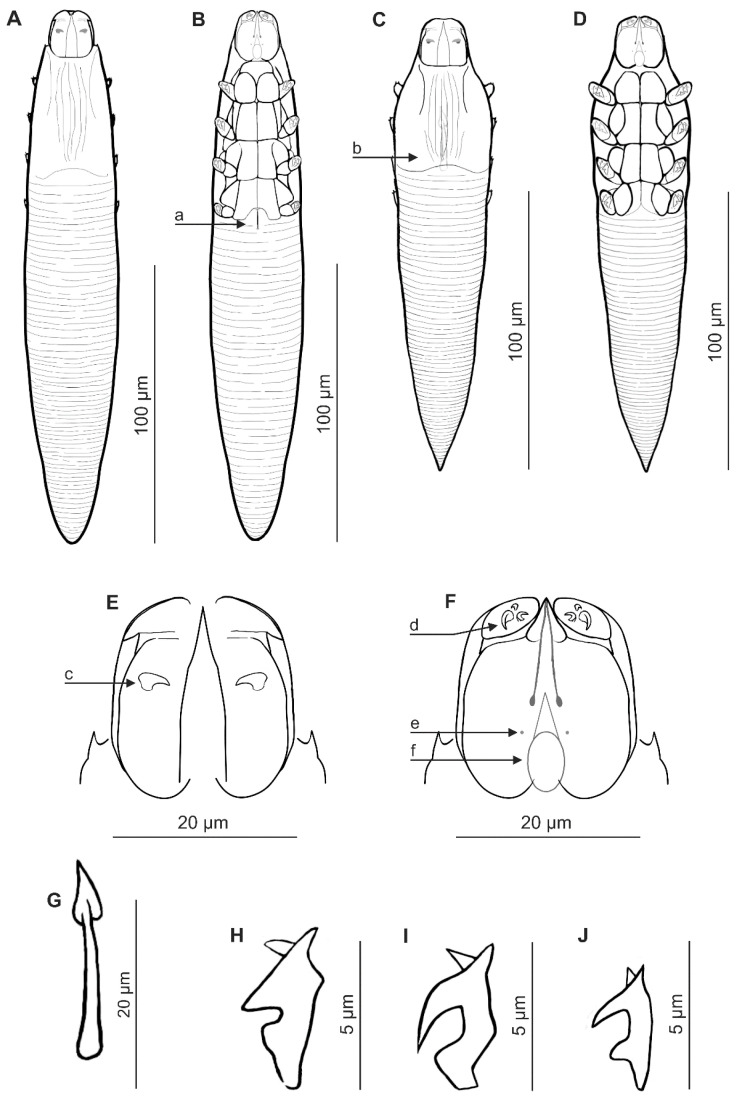
*Demodex procyonis* sp. nov.: female, dorsal view (**A**); female, ventral view (**B**); male, dorsal view (**C**); male, ventral view (**D**); gnathosoma, female, dorsal view (**E**); gnathosoma, female, ventral view (**F**); aedeagus (**G**); claw on the leg (**H**); a: vulva, b: aedeagus, c: supracoxal spine (seta *elc.p*), d: spines on palp, e: subgnathosomal seta (seta *n*), f: pharyngeal bulb, and *Demodex putorii*: claw on the leg (**I**); *Demodex lutrae*: claw on the leg (**J**).

Male (n = 6 paratypes). Conical, shorter than female, 166 (147–185) long, 32 (29–36) wide. Gnathosoma shape similar to that of the female, with length equal to or slightly less than width at base. Pharyngeal bulb and morphological details of gnathosoma similar to those of the female. Shape of podosoma and legs is also similar to that of the female, but all epimeral plates (I–IV pairs) connect medially. Opisthosoma shorter than in females, conical, distinctly tapering towards the end, pointed at the end; it constitutes 53% (53–54%) of body length. Whole opisthosoma distinctly annulated; annuli relatively wide at ca. 3–4. Opisthosomal organ not visible. Aedeagus relatively short, 21 (19–24) long, on the dorsal surface, located between epimeral plates II and III. Genital opening located on the dorsal surface, at the level of the anterior part of epimeral plate II.

Immature stages. Only two deutonymphs were found.

**Figure 2:**
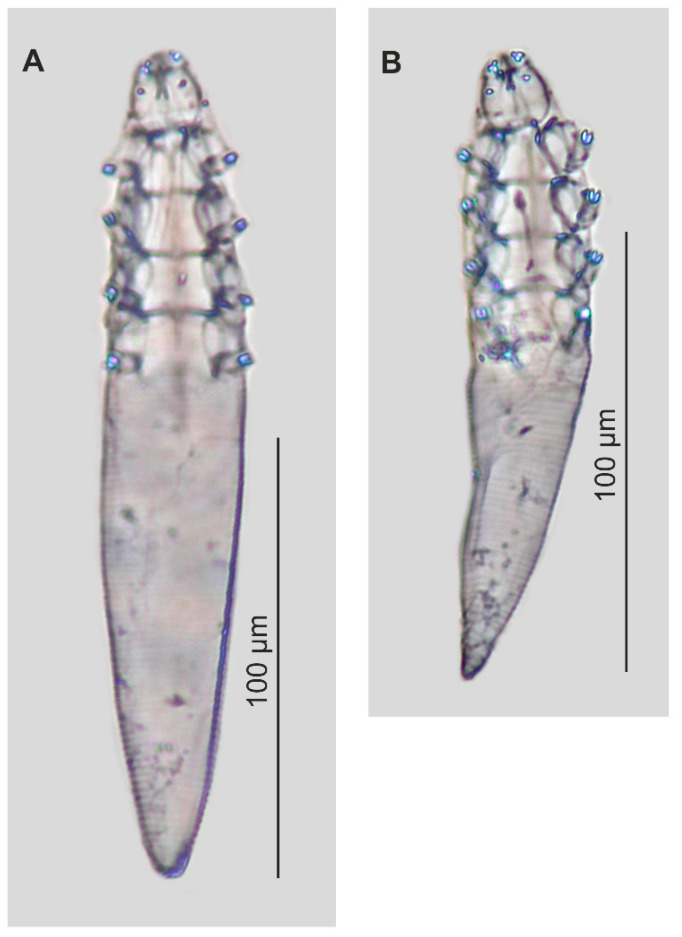
Paratypes of *Demodex procyonis* sp. nov.: female (**A**), male (**B**).

### 3.2. Material Deposition

Holotype female (reg. no. UGDIZPPPlDDp01f), 17 female paratypes (reg. no. UGDIZPPPlDDp02f–18f), 6 male paratypes (reg. no. UGDIZPPPlDDp01m–06m), and two deutonymphs (reg. no. UGDIZPPPlDDp01dn–02dn); skin of the head (cheek), abdomen, back, posterior groin; host *Procyon lotor* (reg. no. MCPPl1/2024, MCPPl2/2024, MCPPl3/2024); Przytok Forest District (Nadodrzański Forest), Poland; November 2024; host coll. D.J. Gwiazdowicz, parasites coll. J.N. Izdebska, L. Rolbiecki, and K. Faleńczyk-Koziróg; the whole-type material (mounted microscope slides with the demodecid mites) deposited within the framework of the Collection of Extant Invertebrates in the Department of Invertebrate Zoology and Parasitology, University of Gdańsk, Poland.

### 3.3. Infection and Location in the Host

*Demodex procyonis* sp. nov. was found in all of the examined *P. lotor*. The mites were found mainly on the head (cheeks, 22 specimens), with some also present on the leg (posterior groin, 1 specimen), back (2 specimens), and abdomen (1 specimen). The observed mites did not cause skin lesions in the examined raccoons. The small number of hosts examined did not allow for an analysis of the population structure of demodecid mites or an assessment of the level of host infestation.

### 3.4. Etymology

The specific epithet *procyonis* refers to the specific name of the host.

### 3.5. Differential Diagnosis

*Demodex procyonis* sp. nov. is a species distinct from other Demodecidae with regard to certain essential taxonomic characteristics for the group, as well as body shape and proportions. Morphologically, it is most similar to species from analogous locations in the European otter, *Lutra lutra* (Linnaeus, 1758) [23] and unpublished data), and the common polecat *Mustela putorius* Linnaeus, 1758 [24]. *Demodex procyonis* sp. nov. is comparable in size to *D. lutrae* Izdebska et Rolbiecki, 2014, and clearly smaller than *D. putorii* Izdebska, Rolbiecki et Rehbein, 2024; has a similarly expressed sexual dimorphism (i.e., males shorter than females), but different body proportions (Table 2). In addition, the opisthosoma of *D. procyonis* sp. nov. females is cylindrical, gradually tapering towards the end, and rounded at the end, while in *D. lutrae* and *D. putorii* females, it is more conical and pointed at the end. The gnathosoma of *D. procyonis* sp. nov. is oval, barrel-shaped, with a length equal to or slightly less than the width at the base, while that of *D. lutrae* and *D. putorii* is trapezoidal, with a length equal to (*D. putorii*) or greater (*D. lutrae*) than the width at the base.

However, these mites differ most clearly with regard to the taxonomically significant structures of the gnathosoma. The supracoxal spines on the gnathosoma in *D. procyonis* sp. nov. are sharpened (pickaxe with a sharp end), 2.5–3.0 long, while in the other species they are smaller (*D. lutrae* 1.5–2.0, *D. putorii* 1.0–1.5) and of a different shape (*D. lutrae*–blunt-ended hammer, *D. putorii*–conical). All mite species possess three spines on the terminal segments of the palpi; however, *D. procyonis* sp. nov. possess two large spines of similar size (including one bifurcated) and one slightly smaller (pointed, unbifurcated, curved), while in *D. lutrae,* two larger and one very small are present (all unbifurcated), and *D. putorii* possesses two larger (including one bifurcated) and one small (conical). The subgnathosomal setae are present on both sides of the pharyngeal bulb but clearly above its anterior edge in *D. procyonis* sp. nov.; in comparison, they are located at the level of the anterior edge of the pharyngeal bulb in *D. putorii* and are located posteriorly in *D. lutrae*.

The structure of the leg elements, including the epimeral plates, also differs. The epimeral plates I–III are well sclerotized, and pair IV is poorly visible in *D. procyonis* sp. nov., while all the plates are well sclerotized in *D. lutrae* and *D. putorii*. Moreover, in *D. lutrae*, the posterior edges of the epimeral plates I–III are very heavily sclerotized. In the female of *D. procyonis* sp. nov., the vulva is situated in an incision between the IV epimeral plates (reaching halfway between these plates); the vulvae in *D. lutrae* and *D. putorii* are present in the same location but not as deeply. The legs also differ regarding the shape of the claws (Figure 1). Furthermore, in all these mites, the aedeagus is located at the level of epimeral plates II and III; however, in *D. procyonis* sp. nov., it is shorter, with the genital orifice located at the level of the anterior part of epimeral plate II; in *D. lutrae* and *D. putorii*, the genital orifice is located at the border between epimeral plates I and II.

## 4. Discussion

Our study of raccoon skin from Poland has identified a new species of the family Demodecidae: the first described from raccoons in Europe. *Demodex procyonis* sp. nov. is distinct from other demodecid mites, including those known from European mammals co-occurring with *Procyon lotor*.

*Demodex* mites have previously been recorded in raccoons only once, within their natural range (Virginia, USA), but their morphological characteristics were not described [15]. The morphological features visible in the published photograph, as well as the size and skin location of the specimens, are consistent with those of the species described herein. It can therefore be assumed that this is a raccoon-specific parasite introduced from its native range. Demodecid mites found in the USA caused disease symptoms in the skin of the head (vibrissal area), but the full topography was not verified at that time [15].

In the present study, the mites were found in all examined raccoons; however, their density was low, and no clinical symptoms were observed. Many demodecid mite infestations in wild mammals are asymptomatic [25]: clinical manifestations are rare and may suggest immunosuppression or poor host condition [26]. In the present study, most demodecid mites were located in the skin of the head, but they were also found in the hairy skin of other body regions of the raccoons. The body shape and location of *D. procyonis* sp. nov. are analogous to those of species inhabiting the hairy skin (hair follicles) of various carnivorous mammals, such as *D. lutrae* from the European otter, *D. phocidi* Desch, Dailey et Tuomi, 2003, from the harbor seal *Phoca vitulina* Linnaeus, 1758, *D. canis* (Leydig, 1859) from the domestic dog *Canis lupus familiaris* Linnaeus, 1758, and *D. cati* Megnin, 1877, from the domestic cat *Felis catus* Linnaeus, 1758 [23,25,27,28]. With regard to the diagnostic characteristics essential for Demodecidae taxonomy, the newly described *D. procyonis* sp. nov. appears most similar to species described from mustelids, such as the otter, the common polecat, and the European badger *Meles meles* (Linnaeus, 1758) [24,29].

The range of expansion of raccoons and their invasions has stimulated parasitological research to determine the role of this host in the circulation of parasites in the environment. Previous studies have confirmed their potential for both introducing new parasites and acquiring local ones [30,31,32,33]. They also indicate that European raccoon populations have a generally good health status, as infections do not lead to parasitic disease. Our current findings represent the first report concerning invasive populations of these mammals in the context of skin mites.

## Figures and Tables

**Table 2:** Morphometric comparison (mean, range and SD) between *Demodex procyonis* sp. nov., *D. lutrae*, and *D. putorii*.

Feature/ Species	*Demodex procyonis* sp. nov.		*Demodex lutrae*		*Demodex putorii*	
Source	Present Study		Izdebska et Rolbiecki [23] and Authors’ Unpublished Data		Izdebska et al. [24]	
Sex Sample Size	Males (n = 6)	Females (n = 18)	Males (n = 24)	Females (n = 76)	Males (n = 40)	Females (n = 100)
Body total length	166 (147–185) ± 18	193 (178–207) ± 9	170 (158–186) ± 9	209 (183–240) ± 12	203 (177–229) ± 14	278 (231–309) ± 16
Body total width	32 (29–36) ± 3	32 (28–36) ± 2	34 (30–38) ± 2	35 (30–41) ± 3	31 (27–35) ± 2	35 (30–41) ± 2
Body length to width ratio	5.1 (5.0–5.3) ± 0.1	6.1 (5.6–7.1) ± 0.5	5.0 (4.4–6.0) ± 0.4	6.0 (4.9–7.1) ± 0.5	6.5 (5.4–8.1) ± 0.5	8.0 (6.8–9.6) ± 0.6
Opisthosoma length to body length ratio (%)	53 (53–54) ± 0.4	59 (56–62) ± 2	59 (57–63) ± 0.02	61 (56–66) ± 0.02	64 (60–68) ± 2	67 (61–70) ± 2
Aedeagus length	21 (19–24) ± 2	–	24 (20–30) ± 2	–	23 (19–27) ± 2	–
Vulva length	–	8 (6–9) ± 1	–	9 (8–14) ± 1	–	9 (5–11) ± 1

## Data Availability

The data presented in this study are available on request from the corresponding author.
